# Loss of control eating during pregnancy is associated with excessive gestational weight gain among individuals with overweight and obesity

**DOI:** 10.1186/s12884-023-05618-1

**Published:** 2023-05-12

**Authors:** Michele D. Levine, Rebecca L. Emery Tavernier, Rachel P. K. Conlon, Jennifer L. Grace, Gina M. Sweeny, Bang Wang, Yu Cheng

**Affiliations:** 1 Department of Psychiatry, University of Pittsburgh School of Medicine, 3811 O’Hara Street, Pittsburgh, PA 15213 USA; 2 Division of Epidemiology and Community Health, School of Public Health, University of Minnesota, Minneapolis, MN USA; 3 Western Psychiatric Hospital, University of Pittsburgh Medical Center, Pittsburgh, PA USA; 4 Department of Statistics, University of Pittsburgh, Pittsburgh, PA USA

**Keywords:** Pregnancy, Gestational weight gain, Loss of control eating, Binge eating

## Abstract

**Background:**

Excessive gestational weight gain (GWG) predicts negative health outcomes among individuals with overweight or obesity. Loss of control eating (LOC), the ingestion of food associated with being unable to control eating, is the core psychopathology of binge eating disorders. We evaluated the contribution of LOC to GWG among pregnant individuals with prepregnancy overweight/obesity.

**Methods:**

In a prospective longitudinal study, individuals with prepregnancy BMI ≥ 25 (*N* = 257) were interviewed monthly to assess LOC and reported demographic, parity, and smoking information. GWG was abstracted from medical records.

**Results:**

Among individuals with prepregnancy overweight/obesity, 39% endorsed LOC prior to or during pregnancy. After adjusting for factors that have previously been linked to GWG, LOC during pregnancy, uniquely predicted higher GWG and greater likelihood of exceeding GWG recommendations. Participants with prenatal LOC gained 3.14 kg (*p* = 0.03) more than did those without LOC during pregnancy and 78.7% (*n* = 48/61) exceeded IOM guidelines for GWG. The frequency of LOC episodes was also associated with greater weight gain.

**Conclusions:**

Prenatal LOC is common among pregnant individuals with overweight/obesity and predicts greater GWG and increased likelihood of exceeding IOM GWG guidelines. LOC may represent a modifiable behavioral mechanism to prevent excessive GWG among individuals at risk for adverse pregnancy outcomes.

## Introduction

Excessive gestational weight gain (GWG) robustly predicts obstetric complications [35], greater postpartum weight retention [28], and adverse neonatal outcomes [8]. Thus, preventing excessive gestational weight gain (GWG) can improve the health of mothers and children. However, more than 45% [17, 37] of pregnant women and people exceed guidelines established by the Institute of Medicine (IOM) for GWG [22]. Compared to those who begin pregnancy with normal weight, those who begin pregnancy with overweight or obesity are more likely to gain excessive gestational weight during pregnancy [28] and retain a larger amount of gestational weight postpartum [1, 45]. To optimize interventions that address excess weight gain, it is critical to identify behaviors related to GWG, particularly among individuals who begin pregnancy with overweight or obesity.

One modifiable mechanism that may contribute to excessive GWG is loss of control eating (LOC). LOC refers to the ingestion of food with an associated experience of being unable to control one’s eating and is the core psychopathology of bulimia nervosa and binge eating disorders [26]. LOC is more common among women than men, affecting up to 30% of women between the ages of 18 and 35 [40]. LOC is associated with increased daily calorie intake, frequent overeating episodes[14, 46] and higher levels of depressive symptoms [9], which themselves are associated with weight gain and obesity [29]. Importantly, LOC is the most frequently reported disordered eating behavior during pregnancy [7, 31, 39]. Although other serious disordered eating behaviors often improve during pregnancy [5, 7, 10], LOC does not improve during pregnancy [7]. To date, rates of LOC during pregnancy range from 8.4 to 36%, depending on the method of assessment and population studied [12, 20, 24, 30]. For example, we previously reported that 24% of pregnant people with overweight or obesity endorsed LOC during pregnancy [24].

In addition to evidence that LOC persists during pregnancy, converging evidence suggests that LOC and binge eating may be associated with excessive GWG [34] although the link to excessive GWG has not been consistently noted [20]. Initial evidence suggested that people who binge eat during pregnancy report more worry about GWG [32, 42], and this worry, in turn, has been associated with higher GWG [21]. Recently, Micali and colleagues [30] found that those who experienced LOC in pregnancy gained an average of 3.74 kg more than those who denied experiencing LOC in pregnancy. Although these findings support a possible link between LOC in pregnancy and elevated risk for higher GWG, previous studies have been limited by the retrospective reports eating behaviors assessed at the end of pregnancy rather than across pregnancy [20, 30], the use of self-reported LOC [20, 21, 30, 32, 34] and the assessment of LOC at only one point during pregnancy [20, 30], limiting the ability to fully characterize the impact of LOC and changes in LOC across the full gestational period on GWG. Moreover, previous studies have been conducted among samples of pregnant people enrolled in interventions designed to promote weight gains within the IOM guidelines [43] or using convenience samples of pregnant people who had not been selected based on risk of aberrant eating or for excessive GWG. The impact of LOC in the context of individuals at highest risk of negative obstetric outcomes and the ways in which LOC may change across pregnancy remain unclear.

Given that LOC is associated with difficulty managing weight over time [18] and tends to persist during pregnancy, we reasoned LOC may be a critical and modifiable component of GWG. Thus, the present study sought to determine the contribution of LOC, frequently assessed across pregnancy and measured using a standard clinical interview adapted for the perinatal period [13] to GWG among people who began pregnancy with overweight or obesity. We hypothesized that LOC during pregnancy would be associated with larger total GWG and with an increased likelihood of exceeding IOM guidelines for GWG. We also predicted that the frequency of LOC episodes during pregnancy would be associated with an increased likelihood of exceeding IOM guidelines for GWG and with larger total GWG.

## Materials and methods

### Participants and procedures

Pregnant individuals (*N* = 257) were recruited from obstetric clinics associated with a large urban hospital. Individuals were eligible if they had a pre-pregnancy body mass index (BMI) ≥ 25 kg/m^2^, were 12–20 weeks gestation, were ≥ 14 years old, and had a singleton pregnancy. Exclusion criteria were use of weight-affecting medications (e.g., steroids) or medical conditions (e.g., thyroid disorders), participation in weight-management programming, type 1 diabetes, or psychiatric symptoms requiring immediate treatment. Note that we did not assess gender identity and thus have selected the more inclusive, gender neutral term, pregnant people to refer to the participants in our study.

**Fig. 1:**
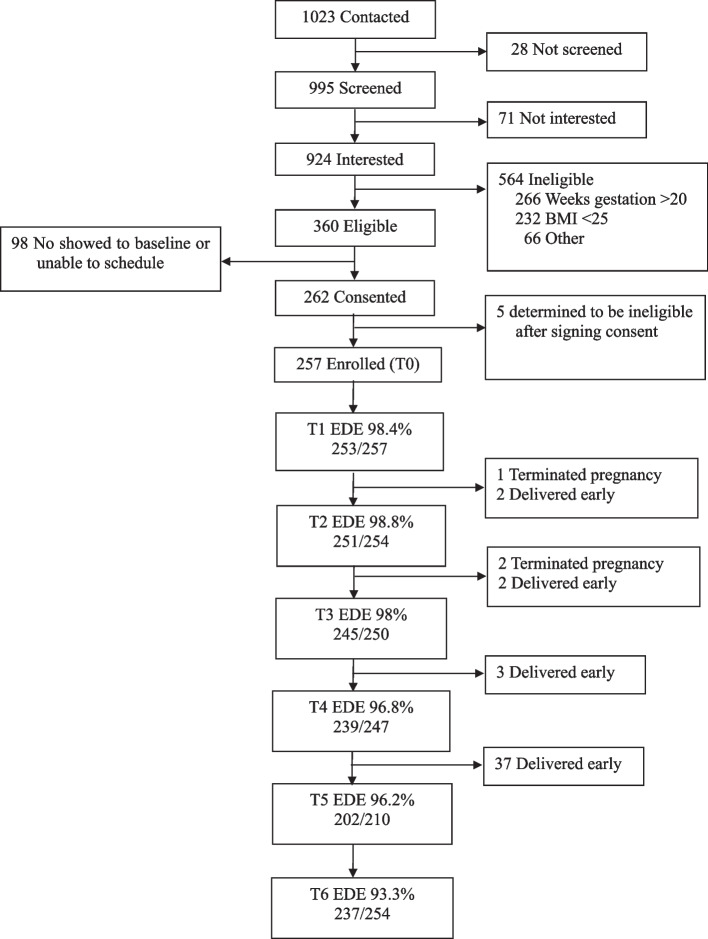
Participant flow and EDE completion rates

As shown in Fig. 1, between September 2012 and January 2017, a total of 1023 individuals were contacted about the study and 995 were screened for eligibility. Those determined to be eligible came for an in-person baseline assessment (T0), during which they completed questionnaires, height and weight measurements, and interviews. Participants then completed 5 monthly telephone interview assessments of LOC (T1-T5). As shown in Fig. 1, at T1, 253 (98.4% of eligible), at T2, 251 (98.8% of eligible), at T3, 245 (98.0% of eligible), at T4, 239 (96.8% of eligible), and at T5, 202 (96.2% of eligible) participants completed monthly interviews as scheduled. A total of 198 participants completed 6 monthly interview assessments of LOC and 59 completed 5 or fewer.

Study procedures were approved by the University of Pittsburgh Institutional Review Board (PRO11070083). Participants 18 years and older provided written informed consent before the initiation of study procedures. Verbal assent was obtained from participants below age 18 (*n* = 4) and written informed consent was provided by a parent or legal guardian. All participants received monetary compensation for their participation.

### Assessments

#### Demographic and clinical information

Participants self-reported demographic information including age, racial identity, annual household income, educational background, and parity at T0. Individuals were asked to describe ethnic identity as either Hispanic or Latina or not and to circle any of the following category or categories to best describe their race: White, Black or African American, Asian, American Indian/Alaska Native, Native Hawaiian or Other Pacific Islander. Given the well established disparity in the health of Black pregnant individuals relative to those of other racial identities [36, 44], we also compared individuals who identified as Black or African American to other racial identities in analyses involving demographic data. Participants also were queried about smoking and smoking status was coded as ever relative to never smoking.

### Pre-pregnancy weight

During the initial phone screen, participants self-reported prepregnancy weight, a commonly used, valid method of obtaining pre-pregnancy weight [38]. During the baseline (T0) assessment, height was measured in person via calibrated stadiometer and weight was measured using a digital scale. Pre-pregnancy BMI was calculated using self-reported pre-pregnancy weight obtained during the initial phone screen and measured height at the T0 assessment.

### Gestational weight gain

GWG was calculated as the measured weight before delivery, obtained from medical records, minus self-reported pre-pregnancy weight obtained during the initial phone screen. GWG was defined as excessive according to IOM guidelines for total end of pregnancy GWG based on pre-pregnancy BMI [22]. Specifically, excessive GWG was defined as a weight gain > 11.3 kg for participants with pre-pregnancy overweight (BMI > 25–30 kg/m2) and > 9.1 kg for those with pre-pregnancy obesity (BMI > 30 kg/m2). Both the dichotomous variable representing GWG above IOM guidelines for pre-pregnancy BMI, and the total amount of GWG were used as dependent variables.

### Medical record data

Because the length of gestation is a potential confounder of GWG, delivery dates were abstracted from maternal medical charts to calculate length of gestation. Data on medical conditions and diagnoses during pregnancy also were abstracted.

### Eating disorder examination–pregnancy version

The Eating Disorder Examination–Pregnancy Version (EDE-PV) is a structured clinical interview designed to assess eating disorder psychopathology [16] modified to capture the unique eating attitudes and behaviors associated with pregnancy [13]. Like the EDE [15, 16], the EDE-PV assesses distinct types of overeating and LOC episodes, including objective bulimic episodes (OBEs; characterized by the consumption of an objectively large amount of food accompanied by a sense of LOC), subjective bulimic episodes (SBEs; an episode of LOC in which the amount of food consumed is not deemed objectively large), and overeating episodes in which LOC is endorsed that occur outside of OBEs and SBEs. As shown in Table 1, we defined any LOC as the experience of any type of LOC episode (i.e., OBE, SBE, or episodes of LOC outside of OBE or SBE).

**Table 1:** Rates of experiencing Loss of Control (LOC) and sample median (interquartile range) of the episodes for those with LOC by LOC types in the three months prior to and during pregnancy

Type of LOC (period of assessment)	Statistics	Prior to pregnancy	T0	T1	T2	T3	T4	T5	Total
		**(3 months)**	**(3 months)**	**(1 month)**	**(1 month)**	**(1 month)**	**(1 month)**	**(1 month)**	
**OBE**	**% (*n*)**	6.9% (17)	10.1% (15)	6.9% (17)	7.3% (18)	4.5% (11)	5.3% (13)	2.8% (7)	23.9% (59)
	**Median (IQR)**	6 (31)	3 (10)	2 (3)	1 (1.75)	2 (1.5)	1 (1)	1 (1)	3 (9.5)
**SBE**	**% (*n*)**	8.1% (20)	15.4% (38)	7.7% (19)	7.3% (18)	6.9% (17)	4% (10)	2.8% (7)	28.3% (70)
	**Median (IQR)**	10.5 (22.75)	2.5 (7.75)	2 (2.5)	2.5 (2.5)	2 (1)	2 (1)	3 (1)	4 (13.75)
**OBE/SBE**	**% (*n*)**	13.4% (33)	22.7% (56)	12.1% (30)	12.6% (31)	11.3% (28)	9.3% (23)	5.3% (13)	37.2% (92)
	**Median (IQR)**	6 (29)	3.5 (7.75)	2 (3)	2 (3)	2 (1.25)	1 (1)	2 (1)	6 (20.25)
**LOC Outside of** **OBE or SBE Episode**	**% (*n*)**	0.4% (1)	1.2% (3)	0.8% (2)	0% (0)	0.8% (2)	0.4% (1)	0.4% (1)	3.6% (9)
	**Median (IQR)**	3 (0)	1 (4)	3.5 (2.5)	0 (0)	1.5 (0.5)	6 (0)	1 (0)	1 (5)
**Any LOC**	**% (*n*)**	13.8% (34)	23.5% (58)	13% (32)	12.6% (31)	12.1% (30)	9.3% (23)	5.7% (14)	38.5% (95)
	**Median (IQR)**	6 (28)	3.5 (7)	2 (3.25)	2 (3)	2 (1)	1 (1)	2 (1.75)	6 (20)

The prior to pregnancy reflects the 3 months prior to participants last menstrual period. T0 reflects the past 3 months, including early pregnancy. T1 -T5 represents past-month assessments during pregnancy. *LOC* Loss of control eating *OBE* Objective bulimic episode, an overeating episode characterized by the consumption of an objectively large amount of food accompanied by a sense of LOC; *SBEs* Subjective bulimic episodes; an episode of LOC in which the amount of food consumed is not deemed objectively large

Interviewers were extensively trained on the EDE-PV. Coding of episode size as well as the presence/absence of LOC episodes were discussed weekly and decisions on size and LOC were made by team consensus. At baseline, participants were queried about the past three months of pregnancy as well as the three months prior to pregnancy, yielding a 6-month recall period as had been used in the EDE version that corresponded to DSM-IV diagnostic criteria [4, 15]. This recall included two distinct measurement time periods relevant to assessing disordered eating pathology during the perinatal period. These two periods (prior to pregnancy and T0 each reflect a 3-month window of time) and the presence (≥ 1 LOC episode) or absence of LOC during these two 3-month periods was documented. At T0, interrater reliability for LOC episodes was high (intraclass correlation coefficient = 0.89) [24].

At monthly prenatal telephone assessments (T1-T5), participants were interviewed using a short version of the EDE-PV, comprised only of the overeating section. The presence (≥ 1 LOC episode) or absence of LOC during each of these monthly periods was documented. Participants were additionally dichotomized according to whether they experienced LOC (≥ 1 LOC episode) at any point during pregnancy (T0-T5).

### Analytic plan

As shown in Fig. 1, participants were excluded from analysis due to stillbirth (*n* = 3) or an inability to obtain medical records (*n* = 7), resulting in an analytic sample of 247 participants. Descriptive statistics were calculated to characterize the sample and study aims were tested using linear and logistic regression. Given that age, racial identity, educational background, income, parity, and smoking status have been linked to GWG [6, 33, 41], these variables were included as covariates in all models. Pre-pregnancy BMI also was included as a covariate in all models given the relationship between pre-pregnancy overweight or obesity and GWG. The length of gestation also is a potential confounder of GWG and was covaried in analyses of GWG.

Analyses were designed to test the association between LOC and total GWG as well as the likelihood of exceeding IOM guidelines for GWG. First, participants were categorized into four groups according to LOC patterns in the three months prior to pregnancy through the end of pregnancy: (a) No LOC prior to or during pregnancy (61.5%); (b) LOC prior to pregnancy but not during (1.2%); (c) LOC during pregnancy only (24.7%); and (d) LOC both prior to and during pregnancy (12.6%). Only 3 (1.2%) participants reported LOC prior to pregnancy but not during and these individuals were excluded from analysis to ensure adequate model fit. Linear regression models were fit to examine the relationship between the remaining three LOC patterns (never, both during and prior to pregnancy, and during pregnancy only) and total GWG, adjusting for the covariates listed above. Binary logistic regression analysis was used to analyze the association between LOC patterns and the likelihood of exceeding IOM guidelines for GWG, adjusting for covariate effects. Comparisons for the linear regression and logistic regression models were respectively tested via t-tests and Likelihood Ratio tests for the corresponding contrasts. We set a priori contrasts between LOC during pregnancy only and absence of LOC prior to or during pregnancy, and between LOC both prior to and during and absence of LOC prior to or during pregnancy.

Next, we used number of LOC episodes in an additional model examining the relationship between LOC eating episodes in pregnancy and GWG. We fit a linear regression model to predict total GWG and a binary logistic regression model to predict the likelihood of exceeding IOM guidelines. Both models were adjusted for the covariates described previously. Because the number of LOC episodes was highly skewed, a natural log transformation was applied to episode data.

Finally, we evaluated the relationship between LOC eating and GWG in the context of obstetric complications that may impact GWG. Two complications were considered as confounders in examining the effect of LOC because of their potential influence on GWG [2]: bed rest (*n* = 2) and diabetes diagnosed during this pregnancy (*n* = 15). Given the low frequency of bed rest (0.8% of the analytic sample), this variable was not further evaluated as a covariate. Diabetes diagnosis during pregnancy was examined in models predicting both total GWG and excessive GWG along with the other covariates. All data were analyzed using SAS 9.4.

## Results

### Rates of LOC

Demographic and clinical characteristics are described in Table 2 as are differences in these characteristics and GWG between those who did and did not experience LOC during pregnancy. Table 1 shows the proportion of pregnant people assessed who experienced an episode of any LOC in the three months evaluated prior to pregnancy as well as in the 5 monthly periods across pregnancy. As shown, 38.5% of pregnant people (*n* = 95) endorsed LOC either prior to or during pregnancy. Thirty-one pregnant people (12.6%) reported LOC both prior to pregnancy and during pregnancy, and 3 pregnant people (1.2%) reported LOC only prior to, but not during, pregnancy. Overall, 24.7% (*n* = 61) endorsed LOC during pregnancy only and did not report LOC prior to pregnancy.

**Table 2:** Sample characteristics (*N* = 247)

Variable	Mean ± *SD* or % (*n*)			
LOC Patterns	All participants	No LOC (*n* = 152)	LOC prior *and* during pregnancy (*n* = 31)	LOC *only* during pregnancy (*n* = 61)
Age, years	28.53 ± 5.42	28.42 ± 5.25	28.86 ± 5.87	28.74 ± 5.79
Racial identity				
Black/African American	45.8% (113)	41.4% (63)	35.5% (11)	60.7% (37)
Others	54.2% (134)	58.6% (89)	64.5% (20)	39.3% (24)
Parity				
Nulliparous	34.8% (86)	40.1% (61)	32.3% (10)	23% (14)
Multiparous	65.2% (161)	59.9% (91)	67.7% (21)	77% (47)
Education				
≤ High School Degree	33.2% (82)	27.6% (42)	35.5% (11)	45.9% (28)
> High School Degree	66.8% (165)	72.4% (110)	64.5% (20)	54.1% (33)
Annual Household Income				
≤ $30,000	65.6% (162)	59.2% (90)	64.5% (20)	82% (50)
> $30,000	34.4% (85)	40.8% (62)	35.5% (11)	18% (11)
Smoking				
No	83% (205)	59.2% (128)	64.5% (24)	82% (50)
Yes	17% (42)	40.8% (24)	35.5% (7)	18% (11)
Pre-pregnancy weight, pounds	197 ± 42.34	193.97 ± 40.77	201.45 ± 45.1	201.61 ± 44.84
Pre-pregnancy BMI, kg/m^2^	32.78 ± 6.35	32.34 ± 6.14	33.97 ± 6.66	33.2 ± 6.78
Pre-pregnancy Weight Status				
Overweight	42.5% (105)	44.7% (68)	38.7% (12)	39.3% (24)
Obese	57.5% (142)	55.3% (84)	61.3% (19)	60.7% (37)
GWG, lbs	13.47 ± 9.34	12.79 ± 8.8	12.56 ± 10.26	15.88 ± 10.05
GWG Category^a^				
Inadequate	16.6% (41)	17.8% (27)	19.4% (6)	13.1% (8)
Adequate	15.4% (38)	16.4% (25)	19.4% (6)	8.2% (5)
Excessive	68.02% (168)	65.2% (100)	61.3% (19)	78.7% (48)
Gestational length, weeks	38.67 ± 2.63	38.38 ± 2.99	39.55 ± 1.63	38.97 ± 1.85

*BMI* Body mass index, *GWG* Gestational weight gain, *LOC* Loss of control eating

^a^Categories determined according to gestational weight gain guidelines from the Institute of Medicine

### Any LOC as a predictor of total GWG

After controlling for relevant covariates, pregnant people who reported LOC during pregnancy gained 3.14 kg more weight than did those who did not experience LOC prior to or during pregnancy (*p* = 0.03; See Table 3). In contrast, participants who reported LOC both prior to and during pregnancy did not differ in their total GWG from those who did not report LOC prior to or during pregnancy (B = -0.60, 95% CI [-8.50, 7.31], *p* = 0.88). In addition, nulliparous pregnant people gained 4.06 kg more during gestation than did multiparous pregnant people (*p* = 0.01). Also, each unit increase of pregnant people’s pre-pregnancy BMI led to 0.2 kg decrease in GWG (*p* = 0.04), after controlling for covariates.

**Table 3:** Relationship of loss of control eating to total and excessive gestational weight gain

	Total GWG, kgs	
Parameter	Estimate [95% CI]	*p*
Intercept	-1.24 [-23.99, 21.51]	0.91
LOC Group		
No LOC (ref)	–	–
LOC prior to and during pregnancy	-0.27 [-3.88, 3.33]	0.88
LOC in pregnancy only	3.14 [0.28, 6]	**0.03**
Age, years	0.2 [-0.05, 0.45]	0.11
Racial identity		
Black (ref)	–	–
Others	-2.09 [-4.96, 0.78]	0.15
Education		
> High School Degree (ref)	–	–
≤ High School Degree	-0.02 [-2.92, 2.87]	0.99
Annual Household Income		
> $30,000 (ref)	–	–
≤ $30,000	0.27 [-3.18, 3.71]	0.88
Parity		
Multiparous (ref)	–	–
Nulliparous	4.05 [1.33, 6.77]	** < 0.01**
Smoking Status at Baseline		
Not smoking (ref)	–	–
Smoking	0.82 [-2.46, 4.1]	0.62
Pre-pregnancy BMI	-0.2 [-0.4, -0.01]	**0.04**
Gestational length, weeks	0.37 [-0.11, 0.84]	0.13

The model examining total GWG was tested using multiple linear regression. *BMI* Body mass index, *CI* Confidence interval, *GWG* Gestational weight gain, *LOC* Loss of control eating

### Any LOC as a predictor of excessive GWG

Overall, 78.7% (*n* = 48/61) of pregnant people who reported LOC during pregnancy exceeded IOM guidelines for GWG. After controlling for relevant covariates, pregnant people who endorsed LOC during pregnancy only (*n* = 61) were more likely to exceed IOM guidelines for GWG compared to those who did not endorse LOC prior to or during pregnancy (odds ratio = 2.36, 95% CI [1.12, 5.26], *p* = 0.03, see Table 3). Pregnant people who reported LOC prior to and during pregnancy (*n* = 31) did not differ in their likelihood of exceeding IOM recommendations for GWG compared to those who did not report LOC prior to or during pregnancy (odds ratio = 0.85, 95% CI [0.36, 2.05], *p* = 0.71).

In addition, pregnant people who were smoking at T0 (odds ratio = 2.48, 95% CI [1.08, 6.11], *p* = 0.04) and nulliparous pregnant people (odds ratio = 2.44, 95% CI [1.23, 5], *p* = 0.01) were more likely to exceed IOM guidelines and gain excessive gestational weight compared to their non-smoking and multiparous counterparts. None of the other covariates were found to be significant in the models of LOC predicting GWG.

### Number of LOC episodes as a predictor of total and excessive GWG

As shown in Table 4, among pregnant people who reported LOC in pregnancy, the log of the number of LOC episodes positively related to total GWG. Pregnant people with more frequent episodes of LOC had higher GWG even after including covariate effects than did those with fewer episodes (2.17 kg, 95% CI [0.52, 3.82], *p* = 0.01). However, in this group of participants who reported LOC in pregnancy, the log of number of LOC episodes was not significantly related to the likelihood of exceeding GWG after controlling for relevant covariates (odds ratio = 1.35, 95% CI [0.89, 2.1], *p* = 0.17).

**Table 4:** Regression models of relationship of loss of control eating episodes to total and excessive gestational weight gain

	Total GWG, kgs	
Parameter	Estimate [95% CI]	*p*
Intercept	-3.97 [-57.63, 49.67]	0.88
LOC episodes, log-transformed	2.17 [0.49, 3.85]	**0.01**
Age, years	0.33 [-0.15, 0.80]	0.17
Racial identity		
Black (ref)	–	–
Others	-1.07 [-6.45, 1.31]	0.69
Education		
> High School Degree (ref)	–	–
≤ High School Degree	3.43 [-1.62, 8.49]	0.18
Annual Household Income		
> $30,000 (ref)	–	–
≤ $30,000	2.96 [-4.19, 10.11]	0.41
Parity		
Multiparous (ref)	–	–
Nulliparous	4.6 [-0.52, 9.74]	0.08
Smoking Status at Baseline		
Not smoking (ref)	–	–
Smoking	-2.21 [-7.97, 3.55]	0.45
Pre-pregnancy BMI	-0.29 [-0.62, 0.03]	0.08
Gestational length, weeks	0.29 [-0.91, 1.5]	0.63

The model examining total GWG was tested using multiple linear regression. *BMI* Body mass index, *CI* Confidence interval, *GWG* Gestational weight gain, *LOC* Loss of control eating

### LOC and GWG after controlling for diabetes diagnosed during pregnancy

Finally, we evaluated the relative contribution of LOC to total GWG and excessive GWG, controlling for the diagnosis of diabetes during pregnancy in addition to the other covariates. Pregnant people who were diagnosed with diabetes during pregnancy (*n* = 15) began pregnancy with a BMI of 40.39 (± 9.21) kg/m^2^, gained 22.04 (± 12.12) kg during pregnancy, and 87% (*n* = 13/15) exceeded IOM guidelines for GWG. Among pregnant people diagnosed with diabetes during pregnancy, the 46.7% who reported at least one episode of LOC endorsed an average of 15.93 (± 30.74) LOC episodes across pregnancy. A diagnosis of diabetes during pregnancy was significantly related to total GWG (*p* < 0.01) and marginally associated with excessive GWG (*p* = 0.09). Importantly, LOC during pregnancy remained significantly associated with both total GWG (*p* = 0.049) and excessive GWG (*p* = 0.04) even after diabetes diagnosed during pregnancy was included as a covariate, though this effect was attenuated.

## Discussion

Among individuals who begin pregnancy with overweight or obesity, 38.5% report loss of control over eating, the core psychopathology of binge eating, either in the 3 months prior to conception or at some point during pregnancy, with 25% of individuals reporting LOC at some point during pregnancy. Individuals who endorse LOC at any point during, but not prior to, pregnancy gain more gestational weight than do those who do not experience LOC. Specifically, pregnant people who report LOC during pregnancy but not in the three months prior to pregnancy gain over 3 kg more than do those who do not experience prenatal LOC. Compared to pregnant people who do not report LOC, pregnant people who report LOC during but not prior to pregnancy are 2.36 times more likely to gain an amount of weight that exceeds that recommended by the IOM based on their prepregnancy weight status. Moreover, prenatal LOC was linked to greater GWG after adjusting for prepregnancy BMI, a consistent and robust predictor of GWG. Thus, LOC is an identifiable behavioral phenotype associated with increased GWG among pregnant people with prepregnancy overweight or obesity. This finding linking LOC to GWG among pregnant people with prepregnancy overweight/obesity is consistent with recent results in a community sample of pregnant people [30]. Micali and colleagues (2018) found that LOC was associated with higher GWG and rates of exceeding IOM GWG guidelines. The current data extend these findings and demonstrate that LOC at any point during pregnancy, is related to greater total GWG and increased likelihood of exceeding IOM recommended GWG amounts among pregnant people with prepregnancy overweight or obesity, a group at high risk for poor obstetric outcomes.

Although LOC was uniquely related to GWG after controlling for factors that have been linked to GWG, parity was also independently related to total GWG, and parity, racial identity and smoking status affected the likelihood of exceeding IOM guidelines for GWG. Nulliparous pregnant people were more likely to have a higher total GWG and to exceed IOM guidelines for GWG compared to multiparous pregnant people. In addition, pregnant people who reported current smoking were more likely to exceed IOM guidelines for GWG relative to those not currently smoking. Previous data suggest that pregnant people who experience LOC during pregnancy are more likely to be multiparous [30], although parity was not a significant predictor in previously reported associations between LOC and GWG [20, 30]. Similarly, previous work has linked smoking cessation to increased GWG [27] though it is important to acknowledge that the 17% of pregnant people in this sample who reported smoking at baseline may have quit smoking during pregnancy.

Although racial identity was not a significant covariate in the models of LOC predicting GWG outcomes, substantial prior research has documented that pregnant people who identify as Black or African American are at increased risk for adverse obstetric outcomes relative to white pregnant people, in part due to systemic factors such as structural racism that impact access to and quality of prenatal care [3, 23, 36, 44]. Relatedly, we have previously documented rates of LOC during pregnancy endorsed by those who identify as Black or African American [11]. Thus, LOC may be a potential target for interventions designed to improve Black perinatal health.

The finding that LOC during pregnancy was independently associated with higher GWG and increased likelihood of exceeding IOM recommendations, suggests the importance of assessing LOC throughout pregnancy. There also are important clinical implications. First, LOC is a potentially modifiable factor that can be addressed during pregnancy. Evidence-based interventions for LOC have been established in non-pregnant individuals, including among those with overweight or obesity [19, 44]. Effective interventions to reduce LOC differ from general weight control interventions and indicate the need to address the psychopathology related to LOC. Previous studies have shown that LOC is prevalent among pregnant people with overweight or obesity, related to weight gain and modifiable. Our results extend these reports and demonstrate that LOC predicts weight gain among pregnant people with overweight or obesity during pregnancy above and beyond demographic and pregnancy-related covariates.

Second, addressing LOC during pregnancy may have important benefits to the offspring of pregnant people who endorse LOC. LOC during pregnancy has been found to predict subsequent eating and weight outcomes among infants and children [30]. Potential mechanisms for this link include biological pathways related to fetal programming, and maternal dietary intake during pregnancy [30], as well as the influence of genetic, epigenetic, and postpartum environmental factors. For example, we previously reported that mothers’ LOC during pregnancy was prospectively associated with obesity-related eating patterns in their infants [25]. Thus, helping pregnant people to manage LOC and eating patterns during pregnancy may have significant benefits for infants and children. Third, it is possible that interventions designed to address LOC will affect not only GWG but also other common perinatal mental health outcomes. Indeed, our work [24] and that of others [20] documents a link between LOC and perinatal depressive symptoms, and it may be that depressive symptoms are a more robust predictor of GWG than self-reported eating patterns [20].

These data are the first from a study specifically designed to prospectively evaluate the relationship between eating patterns and weight gain among pregnant people at high risk for excessive GWG. In addition, the present study used repeated measurements of LOC with a semi-structured interview adapted for administration during pregnancy and data were collected on a large and diverse sample of pregnant people. Moreover, the results considered the contribution of LOC to GWG after adjusting for key covariates. Nonetheless there are important limitations. The study is restricted by the exclusion of pregnant people who do not have prepregnancy overweight or obesity. Additionally, LOC prior to pregnancy was retrospectively characterized at a baseline study visit in the first half of pregnancy. Reporting of eating pathology over the past 6 months may be subject to recall bias, although data suggest consistency in such reports over time [4, 15]. Longitudinal studies that document pregnant people’s LOC and weight trajectories prior to becoming pregnant are warranted to improve our understanding of the course of LOC and its relationship with weight prior to, during, and after pregnancy.

## Conclusion

In summary, results of this longitudinal assessment of eating behavior among pregnant people at high risk for negative obstetric outcomes, suggest that the experience of LOC during pregnancy is related to both higher total GWG and a greater likelihood of exceeding IOM recommendations for GWG and that this association between LOC and GWG remains in the presence of other correlates of excessive gestational weight gain. Together, these results suggest the potential relevance of addressing LOC among pregnant people.

## Data Availability

Data described in the manuscript, code book, and analytic code will be made available upon request.

## Abbreviations

GWG: Gestational Weight Gain
LOC: Loss of Control eating
BMI: Body Mass Index
IOM: Institute of Medicine
T0: Timepoint 0/Baseline
EDE: Eating Disorder Examination
EDE-PV: Eating Disorder Examination-Pregnancy Version
OBEs: Objective bulimic episodes
SBEs: Subjective bulimic episodes
